# Supplementary material for: Caffeine Protects Against Hyperoxia-Induced Structural Lung Injury and Restores Alveolar Development in Neonatal Rats
Source: Antioxidants (Basel). 2025 Dec 12;14(12):1497. doi: 10.3390/antiox14121497 (PMC12729525; doi:10.3390/antiox14121497)
Supplement: Supplementary file 1 [file antioxidants-14-01497-s001.zip › antioxidants-3958589-supplementary.pdf]

Table S1. Sequences of oligonucleotides.

	Oligonucleotide Sequence 5′–3′	Accession No.
	Acta2	
forward	ACCAACTGGGACGACATGGA	NM_031004.2
reverse	CGTTAGCAAGGTCGGATGCT	
probe	6–FAM–CACCACTCCTTCTATAACGA–TAMRA	
	Angpt1	
forward	ACATCCCGTCTTGAAATCCA	NM_053546.1
reverse	TTCCTTGTGTTTTCCCTCCA	
probe	6–FAM–GCTTCTCCAACAGACAAATGAA–TAMRA	
	Angpt2	
forward	TTTGGACCAGACCAGTGAAA	NM_134454.1
reverse	TCCATTAGATCATGCTGCTG	
probe	6–FAM–AAAGAAAGTGCTGGACATGGA–TAMRA	
	Col1a1	
forward	CCCAAGGAGAAGAAGCATGTCT	NM_053304.1
reverse	TCGGAACCTTCGCTTCCATA	
probe	6–FAM–TTTGGAGAGAGCATGACCGA–TAMRA	
	Col3a1	
forward	CCTGGATTCCCGGGTATGA	NM_032085.1
reverse	CTCCCGGAAGACCGTTTTC	
probe	6–FAM–ACACAGAGGCTTTGATGG–TAMRA	
	Csf2	
forward	CATCCAGAGGCCGACATGT	NM_053852.1
reverse	GTCAAGGCGCCATTGAGTTT	
probe	6–FAM–CTGAAGCTATACAAGCAGGG–TAMRA	
	CTGF	
forward	AAAACATTAAGAAGGGCAAAAAGTG	NM_022266.2
reverse	GTGCAGCCAGAAAGCTCAAAC	
probe	6–FAM–TAAAATTGCCAAGCCTGTCA–TAMRA	
	CycD2	
forward	CGTACATGCGCAGGATGGT	NM_199501.1
reverse	AATTCATGGCCAGAGGAAAGAC	
probe	6–FAM–TGGATGCTAGAGGTCTGTGA–TAMRA	
	Edn1	
forward	GCCAGTGTGCTCACCAAAAA	NM_012548.2
reverse	CGCCTTTCTGCATGGTACTTT	
probe	6–FAM–CAGACAAAGAACTCCGAGCC–TAMRA	
	Egr1	
forward	AGCACCTGACCACAGAGTCCTT	NM_012551.2
reverse	GAGAAGCGGCCAGTATAGGTGAT	
probe	6–FAM–CATCGCTCTGAATAACGAGA–TAMRA	
	FGF2	
forward	CGTCAAACTACAGCTCCAAGC	NM_019305.2
reverse	CCATCTTCCTTCATAGCCAGGTA	
probe	6–FAM–AAGAGAGAGGAGTTGTGTCC–TAMRA	
	FGF10	
forward	AGGTCAGCGGGACCAAGAA	NM_012951.1
reverse	TAATGGCTTTGACGGCAACA	
probe	6–FAM–TGTCCGTACAGTATCCTAGAG–TAMRA	
	Fn1	
forward	CCCGGAACAGATGCAATGAT	NM_019143.2
reverse	GGAGCAGGTTCCCTCTGTTG	

	Oligonucleotide Sequence 5′–3′	Accession No.
probe	6–FAM–ACGTCCTACAGAATTGGAGA–TAMRA	
	Grem1	
forward	TGTTACGGCCAGTGCAACTC	NM_019282.3
reverse	TCTTGGGCTTGCAGAAGGA	
probe	6–FAM–CATATCCGAAAAGAGGAAGG–TAMRA	
	HPRT	
forward	GGAAAGAACGTCTTGATTGTTGAA	NM_012583.2
reverse	CCAACACTTCGAGAGGTCCTTTT	
probe	6–FAM–CTTTCCTTGGTCAAGCAGTACAGCCCC-TAMRA	
	PDGFα	
forward	TACCCCGGGAGTTGATCGA	NM_012801.1
reverse	CCCCTACGGAGTCTATCTCCAA	
probe	6–FAM-CTCGAAGTCAGATCCACAGC-TAMRA	
	PDGFRα	
forward	CCTGCGCCGTCTTTAACAAT	NM_012802.1
reverse	TCTCCTCCAGCATGGTGATG	
probe	6–FAM-TTACCCTGGAGAGGTGAGAA-TAMRA	
	PDGFRβ	
forward	CATTCAAGCTGCAGGTCAATGT	NM_031525.1
reverse	CCCCGACAGCGGAGTATCT	
probe	6–FAM-AGTGAGAGTCATCCTGCCAA-TAMRA	
	Pu.1	
forward	GGTGATGGAGACAGCCATAGC	NM_001005892.2
reverse	TGAAGTGGTTCTCAGGGAAGCT	
probe	6–FAM-CACTATTGGGATTTCTCCAC-TAMRA	
	TGFβ	
forward	CCTGCAGAGATTCAAGTCAACTGT	NM_021578.2
reverse	GTCAGCAGCCGGTTACCAA	
probe	6–FAM- TAGCAATTCCTGGCGTT-TAMRA	
	Tie2	
forward	AGGAAGGCCAAAAACAATCC	NM_001105737.1
reverse	TTCGGATGGTGTCCAAGTTT	
probe	6–FAM- TGCCTCCAAAGATGATCACA-TAMRA	
	Timp1	
forward	CGGACCTGGTTATAAGGGCTAA	NM_053819.1
reverse	CGTCGAATCCTTTGAGCATCT	
probe	6–FAM- AGAAATCATCGAGACCACCT-TAMRA	
	Timp2	
forward	GGCAACCCCATCAAGAGGAT	NM_021989.2
reverse	GGGCCGTGTAGATAAATTCGAT	
probe	6–FAM- AGATGTTCAAAGGACCTGAC-TAMRA	
	tPa	
forward	TCAGAAGAGGAGCTCGGTCCTA	NM_013151.2
reverse	TGGGACGTAGCCATGACTGAT	
probe	6–FAM- CAGAGATGAACAGACTCAGA-TAMRA	
	VEGF	
forward	GCTGCACCCACGACAGAAG	NM_001287111.1
reverse	TCTCAATTGGACGGCAATAGC	
probe	6–FAM- AAGTTCATGGACGTCTACCA-TAMRA	
	VEGFR1 (Flt1)	
forward	AATCCCCACAGCAATGTGTT	NM_019306.2
reverse	CGATGCTTCACGCTGATAAA	
probe	6–FAM- TCATTCCGGACTTTCAACAC-TAMRA	

	Oligonucleotide Sequence 5′–3′	Accession No.
	VEGFR2 (Kdr)	
forward	TCAAGCAGCTCGTCATCCTAGA	NM_013062.1
reverse	GGTAGGGTTTCCAGATGTTGGA	
probe	6–FAM- CAGACAACAACCATTGGTGA-TAMRA	

Abbreviations: actin alpha 2, smooth muscle (Acta2), angiopoietin 1/2 (Angpt1/2), collagen type I alpha 1 chain (Col1a1), collagen type III alpha 1 chain (Col3a1), colony stimulating factor 2 (Csf2), Connective tissue growth factor (CTGF), cyclin dependent kinase 2 (CycD2), endothelin 1 (Edn1), early growth response 1 (EGR1), fibroblast growth factor 2/10 (FGF2/10), fibronectin 1 (Fn1), gremlin 1 (Grem1), hypoxanthine-guanine phosphoribosyl-transferase (HPRT), platelet derived growth factor subunit A (PDGFa), platelet derived growth factor receptor alpha/beta (PDGFRα/β), transforming growth factor, beta 1 (TGFβ), TEK receptor tyrosine kinase (Tie2), TIMP metallopeptidase inhibitor ½ (Timp1/2), plasminogen activator, tissue type (tPa), vascular endothelial growth factor A (VEGF), vascular endothelial growth factor receptor 1/2 (VEGFR1/2).
